# Exploring historical trends using taxonomic name metadata

**DOI:** 10.1186/1471-2148-8-144

**Published:** 2008-05-13

**Authors:** Indra Neil Sarkar, Ryan Schenk, Catherine N Norton

**Affiliations:** 1 MBLWHOI Library, Marine Biological Laboratory, Woods Hole, MA, USA

## Abstract

**Background:**

Authority and year information have been attached to taxonomic names since Linnaean times. The systematic structure of taxonomic nomenclature facilitates the ability to develop tools that can be used to explore historical trends that may be associated with taxonomy.

**Results:**

From the over 10.7 million taxonomic names that are part of the uBio system [4], approximately 3 million names were identified to have taxonomic authority information from the years 1750 to 2004. A pipe-delimited file was then generated, organized according to a Linnaean hierarchy and by years from 1750 to 2004, and imported into an Excel workbook. A series of macros were developed to create an Excel-based tool and a complementary Web site to explore the taxonomic data. A cursory and speculative analysis of the data reveals observable trends that may be attributable to significant events that are of both taxonomic (e.g., publishing of key monographs) and societal importance (e.g., world wars). The findings also help quantify the number of taxonomic descriptions that may be made available through digitization initiatives.

**Conclusion:**

Temporal organization of taxonomic data can be used to identify interesting biological epochs relative to historically significant events and ongoing efforts. We have developed an Excel workbook and complementary Web site that enables one to explore taxonomic trends for Linnaean taxonomic groupings, from Kingdoms to Families.

## Background

Taxonomic names represent one of the fundamental tokens that bridge biological knowledge, regardless of its form, across multiple resources [1,2]. The standardized format of *Genus species *associated with a taxonomic name is universally accepted and identifiable across multiple levels of expertise. Furthermore, a well-formed taxonomic name includes a proper name and a four-digit number, which represent the taxonomic authorship and date of description. Efforts such as the Catalogue of Life Program (COLP [3]), an international joint effort to create a single checklist for all known species, are a significant step towards developing common resources to keep track and integrate organism-based biological information across disparate resources.

The Universal Biological Indexer and Organizer (uBio [4]), is a complementary taxonomic information system designed to provide the core services that address name-based impediments to information retrieval [5,6]. In addition to collecting taxonomic and vernacular names from centralized resources such as the aforementioned COLP, scientific names and authority information are also gathered from a range of sources that provide downloads or data transfers. Through the use of natural language processing techniques, organism name, authority, and date information are also extracted from primary literature sources (such as *Nomenclator Zoologicus*). The uBio database of organism names currently exceeds 10.7 million records. Of these records, approximately 3 million are associated with authority and year information.

## Implementation

A series of Ruby scripts were developed to identify and process the requisite data into a form that could be imported Microsoft Excel. First, taxonomic names that contain authority and year information were identified from the uBio NameBank database. Next, the identified names were organized into a unified taxonomic hierarchy that consisted of the Kingdom, Phylum, Class, Order, and Family information inferred from NCBI Taxonomy, ITIS Taxonomy, and Catalogue of Life Taxonomy. The number of taxonomic names associated for each year from 1750 to 2004 was tabulated and output into a pipe-delimited format [see Additional file 1] that could be imported into Microsoft Excel [see Additional file 2]. Searching and graphing features were enabled for the data imported into Excel using series of Visual Basic macros. The data were also loaded into a MySQL database, which was subsequently incorporated into a Ruby on Rails Web application.

**Figure 1:**
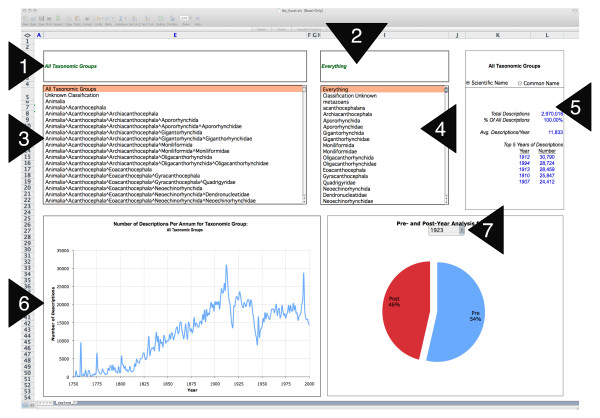
**Browsing taxonomic trends using an Excel workbook**. Users can browse through various taxonomic trends organized by years using an Excel spreadsheet. Briefly, the functionality enable the: (1) Searching of a Linnaean taxonomy down to Families; (2) Searching of common names for selected taxonomic group; (3) Browsing and selection of a Linnean taxonomic group; (4) Browsing and selection of a common name. For the selected taxonomic group, the tool presents descriptive statistics (5); A trend line graph (6); and the number of pre- and post- year descriptions relative to a selected year (7). Only the top half of the Figure is shown; for the full image see Additional file 3.

Through the resulting interfaces (The Excel workbook is shown in Figure 1; details on its availability and use at the supplementary Website [7]) trend-lines can be generated for major super-generic Linnaean taxonomic groups (i.e., Kingdom, Phylum, Class, Order, Family) and also determine the number of pre- or post- descriptions relative to any date between 1750 and 2000.

## Results and discussion

**Figure 2:**
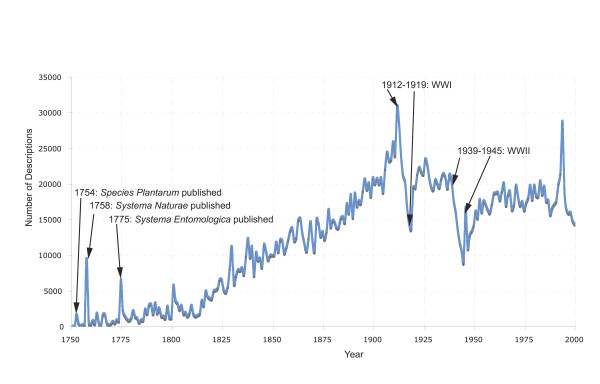
**Trends in taxonomy for all taxonomic groups**. The number of descriptions (y-axis) are shown for every year between 1750 and 2000 (x-axis). Significant historical events that coincide with noticeable changes in year-to-year trends are noted with arrows.

On organizing the taxonomic names from uBio according to their years of description, discernable patterns emerge. As shown in Figure 2, the first three major influxes of names occurred in 1754, 1758, and 1775. These dates can be correlated with the respective publication dates of *Species Plantarum*, *Systema Naturae*, and *Systema Entomologica*.

Two major declines in taxonomy are observed during the periods of 1912–1919 and 1939–1945. Both of these declines are coincident with World Wars I and II. In these periods, the number of taxonomic descriptions falls significantly. Interestingly, while there are large declines in Animal and Plant descriptions, the number of taxonomic descriptions for Bacteria encounters an increase during the same epochs. A speculative hypothesis might be that bacteriology experienced an insurgence during the early 1900's that carried on through subsequent years. This might be reflective of the emergence of methods to study microbes [8]. However, it is important to note that claims of the World Wars directly affecting the rate of taxonomic descriptions would require deeper discussions and historical inquiry of biological/taxonomic activity that is outside the scope of the present discussion.

Following the World Wars, the rate of taxonomic descriptions per year does not reach the pre-World War I peak of 30,790 descriptions until 1994 (28,724 descriptions). Between 1945 and 1994, the number of all taxonomic descriptions per year remains around 18,000. Again, it is difficult to speculate on the actual cause for the last peak in 1994. Some of the cause may be rooted in the quality of the data in the uBio NameBank database, which is a reflection of the data sources wherefrom the taxonomic names originate. For example, the analyzed data indicate that a significant portion of the 1994 peak is due to an increase in descriptions of the family *Cerambycidae*, a large group of beetles. This may very well be reflective of the sources that are used to populate uBio NameBank, which may be more complete with contemporary descriptions of particular taxonomic groups. Thus, we would like to emphasize that it is likely that the last decade of the data analyzed (from 1994 to 2004) may be incomplete, and thus potentially misleading, since there is a delay between taxonomic descriptions and their official vetting and appearance in centralized checklists. A caveat when exploring taxonomic trends and inferring conclusions from these data is that they are dependent on only the currently organized data within the uBio resource. Nonetheless, for the data still likely reflect the general historical trends of data before the 1990's. To this end, names within uBio are dependent on the accuracy and completeness of both expert community checklists and centralized taxonomies. For example, when considering the taxonomic group for butterflies and moths (Lepidoptera), the spreadsheet is biased towards those names that were gathered from LepIndex [9], which is in turn based on a database of a paper-card catalogue that has had few additions since the 1980's (of the ~351,000 descriptions in uBio, ~290,000 are from LepIndex). In addition to continuing to add new checklists and keeping them updated, we are developing natural language processing tools to identify new taxonomic name descriptions as they appear in the literature (e.g., uBioRSS [10]). It is our hope that through navigating the data through exploratory tools, such as the Excel workbook presented here, that additional checklists and other name sources can be identified and shared via resources such as uBio.

Variance in taxonomic nomenclature also accounts for underestimates of some taxa represented in these data, most notably viruses. Because virus nomenclature generally does not follow the Linnaean binomen, author, year format, it can be difficult to automatically infer the author and year for virus taxa. There are plans to identify year information using source data from the ICTVdb [11] and incorporate it into a future version of the dataset.

**Figure 3:**
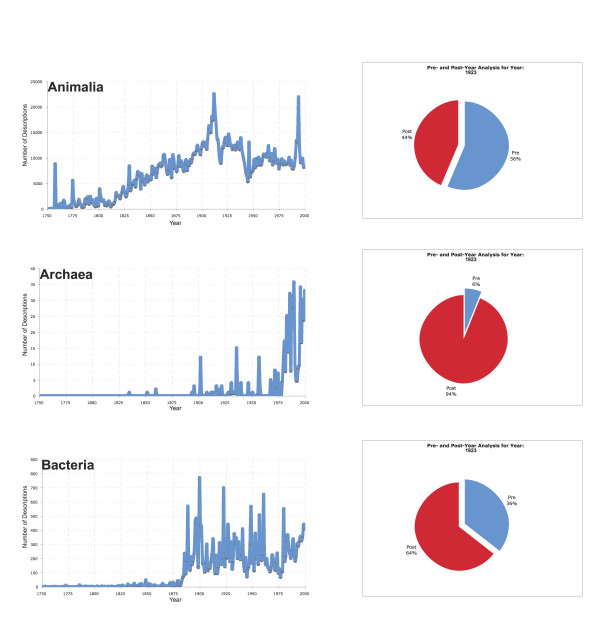
**Historical taxonomic trends, organized by major kingdoms**. The taxonomic trends for each major kingdom are shown in the left column, where the x-axis represents years from 1750 to 2000 and the number of descriptions per year are along the y-axis. In the right column, pie charts are used to illustrate the number of descriptions before (blue) or after (red) 1923, a benchmark date for US Copyright law. Notably missing from the analyzed data are the Viruses, for which dates of descriptions could not be readily identified based on the data in uBio.

Within the context of heritage literature digitization activities, such as the Biodiversity Heritage Library (BHL [12]), the exploration of taxonomic trends for those taxa that are described before a particular date might be useful. For example, an aspect of these types of digitization activities is the determination of how many original descriptions may not publicly be available due to a particular country's copyright laws. Figure 3 shows the number of descriptions from before and after the year 1923 (a benchmark date for copyright in the United States) for each of the major taxonomic kingdoms. It was found that over 1.5 million descriptions (56%) are pre-1923 (excluding 1923); 1.4 million descriptions (46%) are post-1923 (including 1923). It is important to note that the sum of these descriptions (2.9 million) is inflated due to many species that are re-described – for example, *Escherichia coli *was first described in 1895 as "*Bacterium coli*," and then again in 1919 when it was renamed, therefore it is associated with two dates. The can be particularly pronounced in some groups. For example, in the case of the family *Felidae*, the present analysis accounts for 371 names; however, only 40 species of cats are currently known. The present analysis thus suggests that 1.5 million descriptions (or re-descriptions) may be made potentially publicly available in the United States through digitization efforts like the BHL. An interesting future study might also be to compare the number of 'invalid' taxonomic names that are associated with these 1.5 taxonomic descriptions, but can still be linked to contemporary 'valid' taxonomic names. Such a study would involve supplementing the uBio NameBank data with information from current expert-vetted checklists.

## Conclusion

The valuable insights that are possible from analyses such as the ones presented here are due in large part to the meticulous and systematic nomenclature that has been in place for scientific organism names since first suggested by Linnaeus in the 1700's. Here, we have leveraged the systematic annotation of dates associated with scientific names to develop a mechanism to explore taxonomic trends. The resulting Excel workbook and Web interface enable one to explore temporal taxonomic trends for major taxonomic groupings, from Kingdoms to Families.

## Availability and requirements

• Project name: TaxaToy

• Project home page: http://sarkarlab.mbl.edu/taxatoy

• Operating system(s): Excel workbook requires Microsoft Office (Microsoft Windows or Mac OS X); pipe-delimited file can be viewed on any platform; the Web application can be viewed in Web browsers in any operating system

• Programming language: Visual Basic; Ruby on Rails

• Other requirements: Microsoft Excel (for stand-alone browsing of data); Web browser with JavaScript enabled (for Web-based browsing of data)

• License: GNU GPL

• Any restrictions to use by non-academics: None

## Authors' contributions

INS developed the processing scripts in Ruby and the Excel workbook that also involved the development of MS Visual Basic Macros. RS developed and deployed the Ruby on Rails application version. CNN helped with the debugging and design of the Excel workbook. All the authors contributed to the drafting of the manuscript.

## Supplementary Material

Additional file 1 taxonGroupByYears.txt. This file is the pipe-delimited file that was used as the data source for the Excel workbook. It can also be downloaded from the aforementioned website, http://sarkarlab.mbl.edu/taxatoy Click here for file

Additional file 2 tby_Excel.xls. This file can be viewed on recent versions of Microsoft Excel. The workbook requires that macros be enabled. A separate download of this file and additional instructions can be seen at http://sarkarlab.mbl.edu/taxatoy Click here for file

Additional file 3 fig3_taxaByYearsKingdomFigure.pdf. This file contains the full image of Figure 3. Click here for file
