# Supplementary figures and images for: Exploring historical trends using taxonomic name metadata
Source: BMC Evol Biol. 2008 May 13;8:144. doi: 10.1186/1471-2148-8-144 (PMC2408592; doi:10.1186/1471-2148-8-144)

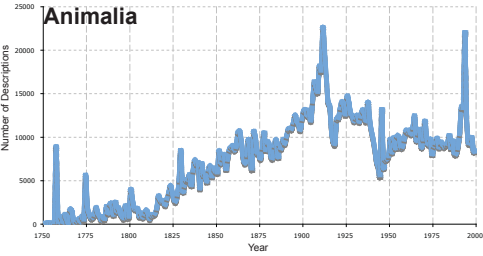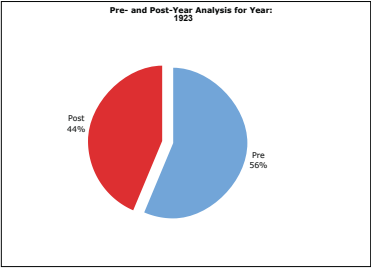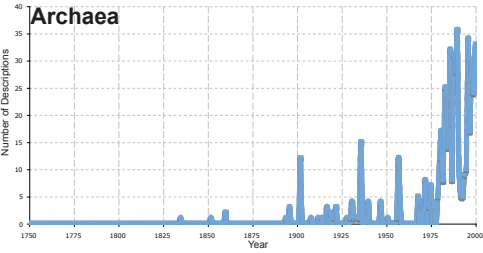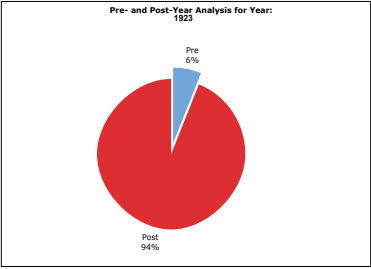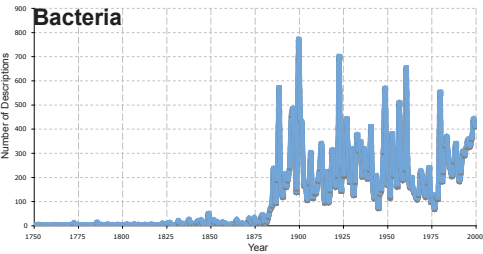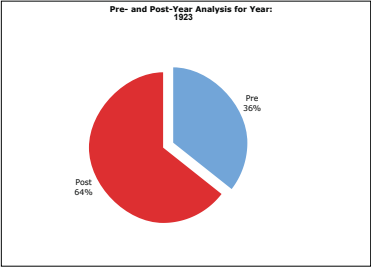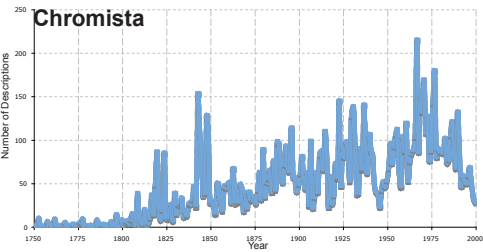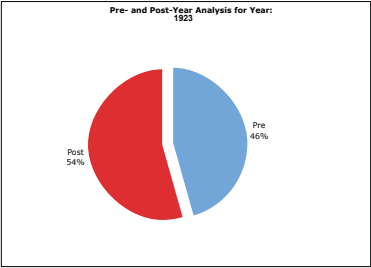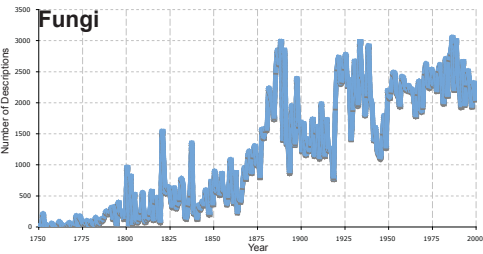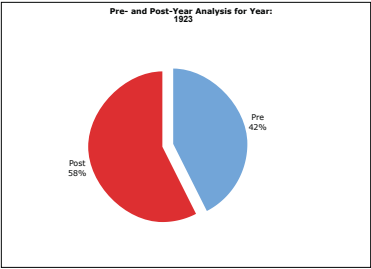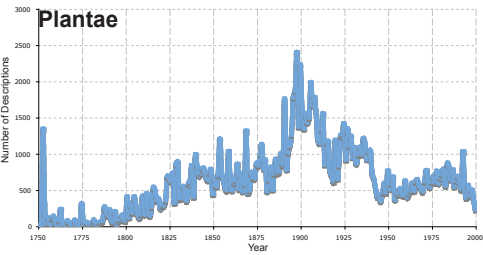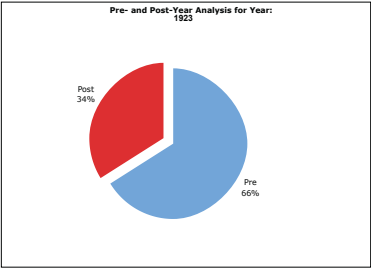

Supplement: Additional file 3 — fig3_taxaByYearsKingdomFigure.pdf. This file contains the full image of Figure 3. [file 1471-2148-8-144-S3.pdf]
